# Inter-generational transnationalism: the impact of refugee backgrounds on second generation

**DOI:** 10.1186/s40878-018-0096-0

**Published:** 2018-10-29

**Authors:** Alice Bloch, Shirin Hirsch

**Affiliations:** 1 Department of Sociology, University of Manchester, Manchester, UK; 2 Department of History, Politics and Philosophy, Manchester Metropolitan University, Manchester, UK

**Keywords:** Community organisations, Inter-generational changes, Political activities, Refugees, Remittances, Second generation, Transnationalism

## Abstract

This paper explores transnational activities among the UK born second generation from three refugee backgrounds: Tamils from Sri Lanka, Kurds from Turkey and Vietnamese. Drawing on qualitative interview data from 45 interviews, the paper explores the views and experiences of the second generation but also their reflections and interpretations of their parent’s histories and transnational activities. The paper takes a comparative and inter-generational approach. It compares transnationalism among second generation with that of the refugee generation and highlights generational differences. The intersections of refugee histories with transnationalism are brought to the forefront of the analysis and in so doing demonstrates the significance of refugee backgrounds on transnational practices.

## Introduction

There is an established literature on transnationalism defining and delineating the activities and behaviours that constitute what is means to be transnational. There is no consensus on the precise definition of the concept, but studies of transnationalism seek to understand how economic, social, cultural and political relations between migrants and subsequent generations shape experiences and practices across time and geographical spaces. For something to be considered transnational the activities have to be regular, stable and resilient overtime (Portes, Guarnizo, & Landolt, 1999). These activities can take place in virtual arenas (Vertovec, 1999) in the country of residence or across international borders. Moreover, transnational activities can take place at different local and national levels and be led by individuals, households, communities and/or the state (Morawska, 2014).

Much of the research and scholarship has focussed on the migrant generation. However over the last 15 years there has been an increasing body of work concerned with second generation, a group that were previously both under-researched and under-theorised (Vertovec, 2001). This emerging literature explores the transnational lives of second generation but there is rarely a specific engagement with transnationalism among those from refugee backgrounds (see for example Crul, Schneider, & Lelie, 2012; Fokkema, Cela, & Ambrosetti, 2013; Kasinitz, Mollenkopf, Waters, & Holdaway, 2008; Levitt & Waters, 2002). Where those from refugee backgrounds are included in studies, they tend to be based on either a single heritage country analysis or multiple countries which include some from refugee backgrounds and some from migrant backgrounds and so the significance of refugee histories is not developed analytically and/or they focus on one component of transnationalism such as remittances or social relations (see for example Barber, 2017; Haikkola, 2011).

This paper fills a gap in the literature by taking a comparative and inter-generational approach that focuses on transnational practices among second generation from refugee backgrounds. Cross border connections can be complex and fluid and link closely to personal biographies. While the focus of this paper is on the views and experiences of the second generation, their reflections and interpretations of the histories and actions of their parents are significant factors in understanding how they frame their own narratives. The paper sets out to answer three questions. First, what are the transnational practices of the second generation and how do they differ from that of the refugee generation? Second, in which ways do refugee histories and current country contexts interact with inter-generational transnationalism? Third, how do social and community networks impact on transnational activities and practices?

## Transnationalism: Refugees and second generation

The concept of transnationalism was mainly operationalized and theorised in relation to labour migrants within the North American context (Koser, 2007). Traditionally research with refugees focused on their refugeehood. However, refugees are also members of families and communities with commitments and obligations that transcend national borders and so share many of the same patterns and characteristics as other migrants in relation to transnationalism. At the individual level, transnationalism can be a function of family ties and these social connections are linked to the sending of remittances and non-monetary gifts (Lindley, 2010). At a macro level nation states can be active in trying to engage diaspora communities and be pro-active in maintaining connections through measures such as voting rights and tax breaks (Gamlen, 2008; Koser, 2007; Délano, 2011).

For those among the refugee generation who envisage returning to the sending country, there is a greater propensity to maintain the transnational ties that will help with reintegration (Bloch, 2008). However for some refugees there is no home to return to and this lack of a viable return, as was the case of Bosnians where their villages and home no longer existed, loosens the ties to the sending countries (see Al Ali, Black, & Koser, 2001). Where there are on-going conflicts, human rights abuses and a lack of democratic representation there can be fear and uncertainly and these influence aspirations for return. Therefore to understand transnationalism it is necessary to reflect on the sending country context (Carling & Pettersen, 2014). This means that the extent and types of transnationalism that refugees participate in will differ between and within national groups and geographical contexts. Rianõ-Alcalá and Goldring’s (2014) research with Colombian refuges in Canada stresses how community formation and transnational political engagement can only be understood in the context of the Colombian conflict. Therefore transnational desire will correlate with the reasons for flight and in some instances the political situation in the sending country. As political situations change so too may the transnational engagement of refugees.

The ability to engage in transnational activities will also be affected by what Al Ali et al. (2001) describe as transnational capabilities. These capabilities relate to resources and can form part of a class analysis of migration and transnationalism where class impacts on the maintenance of linkages (Fresnoza-Flot & Shinozaki, 2017). Experiences in the country of settlement where employment and wages, access to welfare and immigration status (where it is insecure) will all impact on the resources that refugees and migrants have at their disposal to engage in social, political and economic modes of transnationalism (Fresnoza-Flot & Shinozaki, 2017; Hammond, 2013; Rianõ-Alcalá & Goldring, 2014; Vickstrom & Beauchemin, 2016). Unemployment, low income and unsocial working hours are all features of refugees’ experiences in the labour market (Correa-Velez, Barnett, & Gifford, 2015) and this places pressure on both time and the resources necessary for transnational engagement (Rianõ-Alcalá & Goldring, 2014).

While the focus of this paper is on the second generation, any analysis should consider the refugee generation because transnationalism is led by them and because transnational ties are often weaker and less direct among the second generation (Fokkema et al., 2013). Existing research and scholarship shows that the second generation remit less than their parents (Lee, 2011) and this is linked to their age and resources as well as an absence of the close family ties – parents and children - that are associated with remittances (Bloch, 2008; Kasinitz et al., 2008). Where they do remit, rather than sending remittances directly, they might instead engage in fund raising or church donations or the family pool of remittances (Hammond, 2013; Lee, 2011).

Class and regional based inequalities are a factor in transnational engagement (Viruell-Fuentes, 2006) because parent’s economic resources affect the transnationalism of both them and their children. Not only do they affect the quantity and regularity of remittances but they also impact on the ability to travel back to the heritage country (Fresnoza-Flot & Shinozaki, 2017). Visits to study and/or to expose second generation to social and cultural connections and norms help to embed them within transnational social fields (Levitt & Glick Schiller, 2004; Zeitlyn, 2012). Social contact and visits, however infrequent, are symbolic as they can be an important way through which the second generation construct their own ethnic identities within the racialised structures of the country of birth and it can facilitate a sense of belonging and pride in heritages (Viruell-Fuentes, 2006). Home visits can be experienced in different ways and can also alienate where wealth disparities, cultural differences and language deficit are evident (Barber, 2017).

Connections do not, however, rely solely on visits. Social networking sites, or what Lee (2008) terms ‘cyber-transnationalism’ facilitate intradiasporic linkages among the second generation and these are characterized by lateral ties across the globe rather than just vertical ties to the homeland (Baldassar, Pyke, & Ben-Moshe, 2017; Gowricharn, 2009). While appearing to be less transnational, in reality the second generation can be firmly located within transnational ‘webs of connection’, which are global in their orientation (Lee, 2011, p. 295). Significantly, as Baldassar et al. argue, for second generation, ‘diaspora relations are defined by the historical context under which the original migration took place (2017: 946)’. This means that the circumstances of their parent’s migration and their subsequent transnational engagement will influence the transnationalism of their children.

## Sample and fieldwork

The empirical parts of this paper draw on 45 in-depth interviews among the UK born second generation with parents or a parent who had been a refugee from Sri Lanka (Tamil) (16 interviews), Turkey or Northern Cyprus (Kurdish) (14 interviews) and Vietnam (15 interviews). All the interviews were carried out in English. The data this paper draws on forms part of a larger comparative study with France and Switzerland funded by the Swiss Network for International Studies. The comparative element of the fieldwork partly influenced the choice of the three groups included in the study, as there had to be sufficient numbers of each group across the three research sites. However the groups were also chosen because they enabled an examination of the potential impact of different historical and colonial linkages; different reasons for migration; varied religions, ethnicities and community formations. Moreover, the Vietnamese were chosen to explore and compare the long term effects of being part of a refugee resettlement programme - those with refugee status on arrival who were included in a reception programme that offered support with integration but were also dispersed to different geographical locations on arrival – with spontaneous asylum seekers who make a claim for refugee status on arrival in the country of asylum and who may therefore rely on community and voluntary organisations and others from the same ethnic and linguistic group for advice and assistance as was the case among asylum seekers from Sri Lanka and Turkey.

The fieldwork took place in 2014 and 2015 and all the research participants had spent all or most of their childhoods in London. In the absence of a sampling frame, interviewees were located through snowball sampling and volunteer sampling in response to social media posts, through community organisations and key gatekeepers such as local politicians, researchers, colleagues and personal contacts. A range of starting points from which to snowball sample aimed to diversify the networks and experiences though this cannot be measured. The final sample comprised 21 men and 24 women. Ten interviewees were aged 18–20, 15 were aged 21–25 and 20 aged 26 to 36. Those from Vietnamese backgrounds were the oldest group reflecting the earlier arrival of their parents (late 1970s to early 1980s), while Kurds from Turkey or Northern Cyrus were the youngest, again reflecting their parent’s more recent arrival from the early 1990s onwards. The age difference also impacted on their main activity at the time of interview. While 14 of those from Vietnamese backgrounds were working, only five from Kurdish and four from Sri Lankan backgrounds were employed but instead the majority were full-time students.

## Heritage country context and arrival in the UK

Reasons for refugee flight can be significant in relation to state led transnationalism and in terms of the political engagement of refugee and diaspora communities. The majority of Vietnamese refugees in Britain were from North Vietnam and ethnically Chinese having fled Vietnam by boat after the Chinese invasion in 1977 and were resettled in the UK (and elsewhere) after a period of time in refugee camps in Hong Kong (Barber, 2017). A total of 22,577 refugees from South East Asia arrived in the UK between 1975 and 1988 (Sims, 2007). Most had low or no qualifications and no English language similar to the parents of those we interviewed, among whom half had either no formal education or were educated up to primary school.

On arrival they were dispersed around the country as part of a resettlement programme and were often isolated in areas that lacked support structures and access to the labour market (Sims, 2007). As a consequence there was a pattern of secondary migration, from the dispersal areas, to urban centres with pre-existing Chinese and Vietnamese communities that facilitated social networks and access to the labour market (James, 2010). Levels of unemployment were high among the refugee generation. Those who were working were usually in low paid, low or unskilled work, a pattern also found in our research. While the second generation have been upwardly mobile in terms of education (13 have degrees and 2 vocational training) and employment, the relative poverty of their parents would have impacted on the capacity for visits to either Vietnam or to family elsewhere, in what is a very globally spread out diaspora (Baldassar et al., 2017).

Migration from Sri Lanka to the UK occurred in three main waves: affluent Tamils arriving from what was then called Ceylon in the post independence era (1948 onwards), educated middle class students in the 1970s and refugees from different socio-economic backgrounds from the early 1980s (Cowley-Sathiakumar, 2008). During the 1970s there were riots in Sri Lanka accompanied by the emergence of a national consciousness among Tamils, including the formation of the Liberation Tigers of Tamil Eelam (LTTE) who were fighting for a separate Tamil Eelam (state) from 1975. The start of the anti-Tamil progrom in1983 resulted in period of asylum and refugee migration. Moreover, many Tamils from Sri Lanka, who were already in the UK on student visas, feared persecution if they returned and so claimed asylum (Sriskandarajah, 2002).

Tamils have migrated all over the world; the largest single country of settlement has been Canada (400,000). Estimates place the number of Tamils from Sri Lanka in the UK at around 100,000 with most living in what Cowley-Sathiakumar (2008) calls pocket settlements. One area which is particularly notable for its clustering of Tamils from Sri Lanka is East Ham, an area in east London, which has continued to grow numerically as new arrivals from both Sri Lanka and Tamils from elsewhere in Europe were drawn to East Ham by the pre-existing community, kinship networks and places of worship (David, 2008). Among Tamil refugees there is evidence of both ‘place-making’ in London but also orientation – ‘emotional, political and financial’ - towards the establishment of their own state in Sri Lanka (David, 2008, p. 91). The refugee generation, from Sri Lanka, were the most educated and economically successful of the three groups. Half of the mothers and fathers of our interviewees had a degree and that, coupled with their greater propensity to speak English, has led to the acquisition of transferable skills that for some facilitated mobility from unskilled or low skilled work into self-employment or professional jobs.

Like Tamils, Kurds from Turkey also form part of a discriminated against group and this discrimination will impact on the narratives told inter-generationally and on identity (Bloch, 2018; Granata & Sarcinelli, 2012). Kurds from Turkey started arriving in the UK in the 1990s as asylum seekers and estimates place the numbers at around 50,000 with most living in London, clustered alongside the pre-existing Turkish and Northern Cypriot communities. Many found employment in textiles and, following the collapse of the industry, co-ethnically owned small supermarkets and grocery shops (Demir, 2012). Most Kurds from Turkey are Alevis, a religious minority. There is active political organization among Kurds relating to homeland politics with attendance at demonstrations and meetings. There are also active community organisations offering practical support, advice, information and interpreting services to new arrivals. The community organisations continue to have a pivotal role in everyday life and for some are central to their networks. Many Kurds from Turkey come from rural backgrounds and like their Vietnamese counterparts, had few skills that were easily transferable into the UK labour market and little or no English language on arrival (Holgate, Keles, Pollert, & Kumarapan, 2012). Half had no formal education or had been educated up to primary school level and in the UK were employed in predominantly low and unskilled work associated with low pay and unsocial hours.

The paper now turns to the perspectives of the second generation. Although mindful of a seemingly ‘ethnic lens’ approach (Glick Schiller, Çaglar, & Guldbrandsen, 2006) the different histories and backgrounds of the three groups is an essential starting point from which to unravel the complexities of migration, the ancestral homeland and the UK. According to Dahinden it is necessary to follow both ‘a de-nationalized epistemology while simultaneously analysing the potential force of nation-state categories’ (2017, p. 1482) because they can shape identities and the structures that transnational actors are rooted in.

## Scattered families and global social networks

Transnational social networks varied and this depended on where families were geographically located, on technological changes in communications, on return visits to the heritage country and on wider diaspora visits. Phone calls, letter writing and visits were the most common ways of maintaining cross-border social relations and more recently technology has changed the ways in which people connected, facilitating widely dispersed networks. According to Abi,

Before Skype my mum spent so much on those cards, those telephone cards, so my mum would buy one of those like every Friday so we would talk to them and talk to my grandma and aunties…My mum’s social life has gone through the roof because of Skype (Female, Tamil background).

Technology has been significant among the second generation because social media has facilitated connections with more distant family members. While these ties were generally weak and not regular they nevertheless offered a sense of a wider global family and significantly they were sustained without the facilitation of parents.

For some phone calls and then Skype were part of the fabric of family life – they simply happened - but for others there was a sense of inter-generational division where the second generation knew little about their family outside of the UK or in a minority of cases connections were actively blocked. According to Leelong, whose heritage is Vietnamese, there is family in Europe but his ‘mum keeps contact…I have no contact with them whatsoever’. Chi explained how he still had a large number of family members in Vietnam but ‘growing up you didn’t really know they existed’ and Tammi who was 27 at the time of her interview went further feeling that she was actively kept away from her family. She only learned about the existence of relatives when she travelled to Vietnam for the first and only time, aged 21, with her father’s ashes as her mother wanted him to have a Confucius funeral. In the following quote she describes that visit.

So I only met my aunties for the first time when I was 21, before that I didn't even know they existed. So I went back to Vietnam because we were bringing my dad's ashes back, literally got off the boat, got into a car and this man and this woman were there, and they just turned round and went 'oh by the way we're your aunties and uncles' and I was so shocked, I'd never heard anything about them before in my entire life. And I was like oh my gosh they have the same face as me, this is really weird.

Tammi felt her parent’s had made the choice to keep her separate from anything and anyone in Vietnam because, ‘there was so much politics in Vietnam still, as a Communist country…it’s a very dangerous country’. The consequence was a feeling of rupture from Vietnam and frustration with her mother because, she says, ‘I can’t get through the Vietnamese doors, she keeps them closed, slam shut’.

**Fig. 1:**
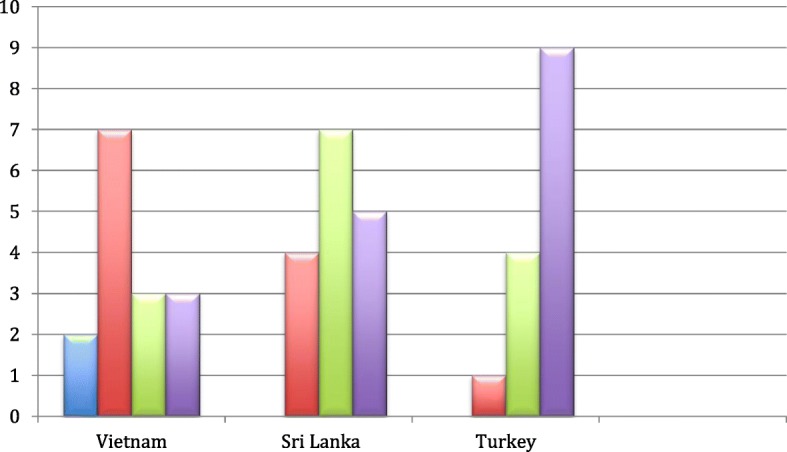
Visits made to the heritage country. Column 1: No visits. Column 2: 1–2 visits. Column 3: 2–4 visits. Column 4: More than 4 visits

All three groups were globally scattered and this dispersal could influence the nature of social relations and the resources needed for visits. Figure 1 shows visits made by our interviews to the heritage country.

Those from Vietnamese backgrounds had made the fewest visits and two had not yet been to Vietnam. Visits did make a difference. Those that went back as adults, for the first time, felt much more conflicted and alienated by the experience than those who had made repeated visits to the heritage country as children. Leelong made his first and only trip to Vietnam aged 20 and describes the experience in the following way,

…going back was a heavy experience. And also meeting 30 40 people relatives cousins or aunties I don't know the first thing about them but they knew everything about me. So yeah it just felt really bizarre. I also kind of felt zero connection with these people which was quite sad but at the same time I remember sitting with my cousins and having zero things to talk about just nothing. I could speak Vietnamese but just basic things meaningless chit chat no connection at all. So going there and then coming back it made me feel a bit funny really.

Making regular visits as children resulted in a greater connection where the heritage country was described as ‘back home’ and ‘home’ although most were aware and commented on the differences between the UK and the ancestral homeland and were also conscious that they were perceived and felt ‘like a you’re a tourist in a country you’re from’ (Leyla, Female, Kurdish heritage). Others, who had made annual or regular visits as children, recalled being ‘bored’ simply staying at the family home in the village and as adults had opted not to return at all or less frequently, as Anton explained.

Every time there was peace talks happening we'd go. My dad loves the holidays he works all the time then he can go Sri Lanka. I don't wanna go no more though, I like the country I like Jaffna, I love everything about it, but I wanna go see other places, like maybe Australia, I've been there [Sri Lanka] way too many times now. My dad likes just going to people's houses and chilling, that's how it is just talking and going to people's houses, I find that boring now (Anton, Male).

The heritage country context did make a difference both in terms of making visits, the places that were visited and the experiences during those visits. These visits were affected by refugee backgrounds and so impacted on second generation in particular ways that were specific to the national context. For those from Turkey the experience of return visits highlighted the discrimination of Kurdish people. Some experienced direct racism and there was an awareness of needing to adjust language and behaviour in the public domain. According to Agir his family,

…wouldn’t speak Kurdish openly in the road…they wouldn’t openly call my name out loud cos I’m named after a sort of like a commander of the PKK…if they were to call me that in a place like this in Turkey, everyone would just give dirty looks.

In the following quote Rodja explained how being from a Kurdish background affected her experiences.

When I go to Turkey they check my passport and read my name and I say ‘yeah, I’m Kurdish’. It’s 2015, there is something fundamentally wrong when I am still questioned about my name…I hate that place…Every year I go there and I still get indirect discrimination.

Among those from Sri Lankan backgrounds, the civil had impacted on their visits but they had also been an important aspect of building connections to Sri Lanka as a place but also to their families.

I went Sri Lanka in 2004 for the first time…during the war, but there was a ceasefire. So I went then. I got to know more family there, so we got to see them then…We went Jaffna...since then my ties to Sri Lanka strengthened (Abhimanyu, Male)

During the civil war it was hard to grasp where you were actually from because I would only be allowed to visit the South, so it was hard to imagine what the North was like. But after the war I went to the North…when I go back I feel like I’m going home. Because I’ve got family there, everyone speaks Tamil there, your culture is there, so it’s quite nice (Krishnan, Male).

While those from Vietnam had made fewer visits, as noted above, the visits could be significant offering an opportunity for story telling and sharing of pre-migration experiences. John for example, who knew little about his parent’s lives in Vietnam, described a significant moment with his father on a return visit.

I was in a car with him when we were in Vietnam, we were just driving around and we were driving over this bridge and he was telling me when he was younger, this just all got bombed. And it was so much detail, he was going into so much detail.

While second generation are less embedded than their parents in everyday social connections the return visits – or decisions not to visit - could be significant in identify formation by creating an understanding of the pre-migration experiences of parents and in raising consciousness about the impacts of conflict and discrimination.

## Sending remittances

Financial contributions to charities, political groups, for post-conflict re-construction or development projects by the refugee generation were limited. Instead most economic engagement was in the form of financial or other remittances sent or given directly to family members when making return visits. The second generation perceived their parent’s remittances in different ways. For some, especially those from Sri Lanka, it was understood as an obligation to the extended family, given the financial disparities and the culturally embedded expectations. As Panya pointed out, ‘small money here is big money there’. He went on to explain how sending money was part of the reason for his parent’s migration in the first place and so sending money was ‘seen as just their dues’. However as Abi explained, framing her narrative within the context of the civil war in Sri Lanka, this was not without cost for her parents.

...they were so desperate for money in Sri Lanka and because he [father] was the only guy in the family they depended on him. The fact that war was going on, everything got destroyed my grandma’s house, the roof; there wasn’t even a roof in the house because of the war and bombing. So they had to work so much…because she [mother] has two younger sisters and they were still in education, my mum had to pay for their university. So they didn’t have that much enjoyment in life because it was more about family and paying to support the family (Female, Tamil background).

While Abi suggests that sending remittances, regardless of the costs was simply how it was, a minority expressed an element of resentment due to what they perceived as the unreasonable expectations. According to Kim those in Vietnam see relatives from London as ‘a suitcase full of cash’. In fact the expectations were perceived to be so high among those in Vietnam that Anne’s parents had not made a return visit for 20 years because they simply could not meet the financial demands as her father had health problems and did not work and her mother worked in the take-away shop owned by her brother. According to Anne,

…you're expected to show money and when I say money I'm not talking like a few hundred like I mean you have to show the big money. Cos they think that you're in a rich country…it's not really like that for my parents so it's difficult.

Remittances were not always expected but could be more informal and ad hoc. According to Alia, her parents ‘didn’t send a big amount of money, but just what they were able to’. Agir also explained how the relative wealth of his family in the UK compared to those in Turkey was important and so,

Anyone who goes back to Turkey on holiday, they would give some money, to give back…Cos they’re better off, they’re not completely well off, but they’re just better off.

There was a clear generational shift among our interviewees. Occasional gifts aside, the second generation were not economically involved and there was no evidence of regular financial support. Rachel explains the generational differences between her and her parents in the following quote.

He [father] would always send money over to his mother. That’s why they have a go at me, because basically their attitude is once you start earning then you give your money to your parents and that’s what you do. Whereas me, I probably spent my money on drinking and going out clubbing with my friends...They worked really hard, sometimes my dad had three jobs at the same time and always sending money back to Sri Lanka.

Other research has also shown diminishing economic engagement by generation. Where the second generation do make financial contributions, it is often part of a household or community one and driven by the parents or community based organisations and may include charity or fund raising events (Hammond, 2013; Lee, 2011). Sending states try to maintain connections with migrants incentivizing on-going engagement through strategies such as tax breaks, voting rights and dual citizenship (Gamlen, 2008) but there is a longer term issue of how to ensure that the second generation are economically committed given the importance of remittances to some sending country economies (Lee, 2011).

## Social, cultural and faith activities: Transnationalism at home

Social and community networks were part of the everyday lives of many of those we interviewed, and their families, when growing up. This varied by proximity to co-ethnic networks and so those living outside of geographical clusters were not involved in community organisations as there was less immediate access to organsiations as Paul explained.

Well the big Vietnamese community was basically in Hackney and some in South East London but not where I lived in East London in Newham. So it was not a big community and we were not connected.

Geography was not the only determinant of community engagement. Some from the refugee generation had made a conscious decision to remain separate from community organisations and networks from the same heritage group. To understand these differences it is necessary to also reflect on pre-migration experiences and/or political positioning among the refugee generation. For example among some Tamil heritage families, where they had different political views, the strategy was separation from the wider community as Vanan explained.

Quite a lot of the Tamils here were Tamil Tiger friendly whereas my parents were staunchly anti-Tamil Tiger… my parents made an effort actually to distance us with the Tamil community (Male, Tamil background).

Modes of community engagement tended to be different among the three groups. While only a minority of the refugee generation from Vietnamese backgrounds participated in formal community organisations they almost always sent their children to Vietnamese or Chinese language schools and families had networks of friends who were also from Vietnam. In contrast those from Kurdish backgrounds rarely went to language schools but were, as families, activity involved in community centres. Aram and others talked about a community centre where ‘every Kurd goes’. Some of those from Kurdish backgrounds had maintained or re-connected with community centres into young adulthood. Zelat for example had attended the Alevi centre as a child then stopped going until she was 16 but is now very involved.

When I was 16 I started to come by myself to youth meetings and we started youth projects and I’ve been on the youth committee for a long time…I feel connected herewith the Kurdish community (Female, Kurdish background).

For the refugee generation community groups were often the first place they went to as they offered information, advice, support and even practical help such as a place to sleep and food. They were also closely linked to political groups among Kurds from Turkey and so community centres had social, cultural, educational and political functions for those who used them.

As a young child we used to go to X [name removed], which is a community centre. We used to go on the marches, free Kurdistan, free our Kurdish leader who was captured in I think it was 98 or 97, and yeah we used to go there quite a lot and my uncle was part of the folk dance ….I used to hang out with a lot of my cousins there because their families used to come and we’d all just hang out (Ezgi, Female, Kurdish background).

While many of the Kurdish community centres were thriving, there was a sense that the Vietnamese community centres that had existed in the cluster areas of south east London and Hackney (east London) were closing. Leelong grew up in Peckham, a neighbourhood in south east London where their parent’s social lives were exclusively with other Vietnamese people in the locality.

…my parents till this day they throw parties have people come round with big banquets of Vietnamese food, sing karaoke, Vietnamese bloody karaoke…my parent's friends would come over and bring their kids so we'd all be in a room hanging out, all Vietnamese kids.

Leelong went to language school at a local community centre. In the following quote he observes the rapid change in the centre.

When I was younger I went to Vietnamese school at the weekend, every Saturday. It's quite bizarre I've got an older brother…he's only seven years older than me, but this community centre was a thriving place everyone went in Peckham, there's a massive Vietnamese community there, and everyone would go there it was thriving there. But by the time I went there it was half the people who went, or less….I'd learn Vietnamese or just play pool with everyone or just kind of sort of hang out, or try and learn about Vietnamese culture. I haven't been in ages now but if I'm correct in what my mum is telling me about it it's pretty much died this centre which is a bit of a shame.

Formal religion played a role in the lives of those from Tamil backgrounds. Regardless of their current practices, as children places of worship, rather than community centres, had been central to their community lives and social networks. Saguna observed how ‘a lot of Sri Lankan Tamils are quite religious’ (Female, Tamil background). According to Samma, a young woman aged 22 at the time of her interview, growing up Hindu meant ‘going to Temples all the time’, and the temples were, ‘just for the Tamil Sri Lankans really’. Janith whose parents attended the Ceylon Pentecostal Church noted that, ‘church was the main institution. I don’t think there were any community centres’ (Male, Tamil heritage).

Although many second generation Tamils still went to a temple or church, it was less frequently than their parents and tended instead to be for major festivals. Abi explained how her generation tended to engage with community differently focusing more on culture than politics.

I think people are more forward thinking in terms of it’s not about Tamils just having their own country now it’s about Tamils living together and being part of something…But I guess the community plays a bigger role in the younger generation especially with the language, culture, dance, music. I think we embrace it more than the older generation.

Among the Kurds from Turkey there was very little formal religious activity – though for Rojda being Alevi was a significant part of her identity because for her it symbolized the oppression of Kurds in Turkey. She described her parents as indoctrinated but also fearful and unable to express or identify as Alevi as a consequence.

When my parents were growing up in Turkey I think you have an idea of Alevism, we aren’t part of Sunni or Islam. When they were growing up, during Ramadan, they wouldn’t need to fast, they were forced to. Forced to fit in. They learned Islam in compulsory religious education about the glorious Islam and Islamic empire.

However for most of those from Kurdish backgrounds religion and religious identity were not central to their lives.

I guess most Turkish families aren’t very religious, because they’re not very strict Muslims. They were Alevi Muslims. You don’t fast, you don’t go to the mosque (Leyla, Female, Kurdish background).

However, Kurds from Turkey were most active politically and this activity had been part of their childhoods transferred through the community centres and families, as the next section explores.

## Political transnationalism and heritage country engagement

Among our interviewees a close link between their parent’s reasons for flight, their political activity and the wider family context was evident in determining political transnational engagement though the correlation was not uni-directional. In some instances parents who had been politically active prior to exile continued to be active in the UK while others chose not to be and even distanced themselves from people and places where there would be different political positions or because they had experienced trauma and wanted distance. Political engagement and political positions were dependent on individual biographies and these affect inter-generational political engagement.

Vietnamese families were not involved in political activities and there was a tendency for interviewee’s parents to close down any discussions of their pre-migration experiences. According to Giang, those who were ethnically Chinese were, ‘very bitter about the country and don’t like talking about it (Male, Vietnamese background). The one area that emerged among a few of those from Vietnam that we interviewed was the divide between the north and the south. Almost everyone we interviewed was from the north. Emphasising this difference Paul described his mother as ‘South Vietnamese, so she’s the enemy basically, haha I’m joking’. Kim’s family were also from the south and had lost all their wealth in the communist invasion including the family home that became an army head quarters. She explained how from childhood, the fact that she was from the south was ‘ingrained’ and that ‘northern people were different’. Regardless of these divisions, they did not transcend borders or political activities but instead formed part of the identity of the second generation.

Among those from Sri Lanka, there were political divisions between those who supported the Tamil Tigers and the separatist movement more generally and those who did not or were no longer engaged in politics. While in theory the civil war in Sri Lanka had ended in 2009 – during the early or mid teenage years of most of our interviewees - in practice that did not mean resolution. While second generation were generally not politically active there was a growing interest in politics as they reached adulthood. There seemed to be a generational division.

There’s this idea that that [politics] was the preserve of the first generation and the second generation are meant to get on with their own lives (Kaliban, Male, Tamil background).

His parents had been very politically active in Sri Lanka but had, according to Kaliban become ‘disillusioned with the Tamil separatist movement’ as his aunt had been killed by the Tamil Tigers and this influenced their lives in the UK where they avoided community activities where they might encounter those with different political views. Vanan’s family were also separate from community politics and in fact the community in general distancing themselves, like Kalban’s family, partly due to political reasons but also because of the family trauma that led to exile.

My mum left because her brother was politically active. He was murdered by the Tamil Tigers. That's why she was very anti Tamil Tigers. And that's also why my parents weren't part of a Tamil community here (Vanan, Male, Tamil background).

Krishnan’s parents did not talk to him about Sri Lanka, saying how ‘they just wanted to get on with their life here’ so instead he found out about the conflict through friends and what he described as politically active ‘older brothers’ from the temple. He had become more interested in politics and in Sri Lanka as he reached his teenage years and described himself as ‘always in the loop’ in terms of talking to his peer group about Sri Lanka. In the following quote Krishnan highlights how his feelings differ from his parents.

For my parents, they’ve been through it and they want to move on whereas for me, I’m angry about it. I feel like my parents didn’t deserve this. They need their justice…I do want to do something because of what’s happened to my parents and everyone who had to move away from Sri Lanka.

Where families were distanced from larger community clusters and community organisation either by choice or through the circumstances of geography it did impact on second generation. Kurds from Turkey were the most recently arrived refugees among the three heritage groups and both the refugee generation and the British born second generation had either been or continued to be active politically, even if only a participant in the occasional demonstration. Their activism reflected the on-going situation in Turkey and the centrality of community organisations as sites of activism and engagement. Community organisations displayed posters about marches, Kurdish political prisoners and campaigns. Political consciousness was also raised through conversations with family members about life in Turkey and by going on demonstrations and protests with parents. For some politics was simply inherited, passed on from parents to their children where there was an unquestioned expectation that they would be involved, as Alia explained.

…that’s what you should be doing, cos you’re Kurdish, you’re people are being oppressed, you should be going to protests, standing up for them (Female, Kurdish background).

Most from Kurdish backgrounds had attended demonstrations in London at some point during their childhoods and they were remembered fondly. Ezgi described how her family brought picnics to the park, along with their placards to listen to the political speeches while for Didem ‘it was a fun day out’.

According to Gilay, politics ‘is something I’ve just grown up with…it’s kind of just stayed with us’. However, while she has become more politically active her father has in contrast become less active and has passed the political baton inter-generationally.

My dad, he’s much less politically active than when he was younger. I guess now he sees his job is to just work and just help pay for me and my sister’s tuition fees. That’s really sad, I don’t want him to feel like he has to work all the time but he does. He’s kind of passed it down to me and my sister so we’re always involved now…political stuff and Kurdish stuff like demos, student protests (Gilay, Female, Kurdish background).

In contrast though was Leyla’s father, who had also been politically active in Turkey and had been a political prisoner. During her interview Leyla described scars on her father’s back that were the evidence of torture. Once in the UK her father orientated himself away from politics and towards family life. Leyla’s mother was pregnant with her when she arrived in the UK under family reunion. They cut political ties but also had clear ideas about what they wanted for their children.

For my parents their first concern and priority was to survive and feed themselves and look after their kids here. And because they had kids they took a back seat...he didn’t want us to be involved. He spent time in jail for protesting and I don’t think he wanted the same thing for his kids.

For Leyla growing up, in an area of London that she described as ‘a very white middle class / working class environment’ where her friends were ‘white’ she identified London as home observing that, ‘it just happens to be that our parents are Turkish’. She was aware how the part of the city she lived in determined so much of her identity and community engagement and it was a huge contrast to others who attended the community centres and political protests.

Like if I grew up in Hackney it’d be different. Or especially around Haringey it’s like little Turkey, you go to school with Turkish people, you shop in Turkish shops, you talk in Turkish.

It is clear that among both the refugee generation and the second generation histories affect political transnationalism in different ways. Moreover, the on-going political situation in the heritage country as well as decisions to be connected or separate from others from the same heritage group also had an inter-generational impact on political engagement.

## Conclusion

This paper contributes to the growing literature on second generation through its comparative analysis of different refugee heritage groups and on the generational shifts in transnationalism. The paper has highlighted the impact of parent’s histories and the heritage country context on different modes of transnationalism, which was most evident in relation to political activities but also intersected with the participation in social, community and cultural activities. Some were embedded within communities while others consciously distanced themselves depending on experiences, aspirations and political positions. The second generation from refugee backgrounds share some similarities with others from non-refugee backgrounds: fewer social transnational connections of less intensity than the migrant / refugee generation and little economic engagement. Where they differ and what is specific to those from refugee backgrounds are the ways in which return visits and political engagement can be affected by the specificity of the heritage country context.

Living in cluster areas, where there are community centres, community places of worship, family, friends and wider social contacts results in greater political consciousness and a stronger identification with the heritage country. For some, politics was a central part of family and community life absorbed through family narratives, discussions with friends and through everyday practices in community based organisations. Those from Kurdish backgrounds were most embedded in community and political activities and these activities were usually linked. Community centres had formed the fabric of everyday lives for many of their parents on arrival to the UK and for the second generation growing up. For those from Tamil backgrounds, places of worship were the loci of community activities but overtime these had become less central in the lives of second generation.

For those living outside of community clusters there was a different and usually more distant identification with community and with politics and this could be a conscious decision by parents to separate themselves as a consequence of previous political activities or political differences with the majority. However, social and cultural linkages and political consciousness was not only raised in the UK context, the geographical restrictions (e.g. not visiting Jafna during the civil war) and experiencing racism (Kurds in Turkey) during visits to the heritage country reinforced the specificity of the refugee context that led to their parent’s migration. Awareness of these experiences have shaped the transnational perspectives and engagement of the second generation from refugee backgrounds in ways that can set them part from those from non-refugee backgrounds. The national context does make a difference but so too do the more micro aspects of biographies and family relations. Reaching adulthood offers new modes of engagement separate from families but more focused around peer group with proximity making a difference.

The paper has highlighted the complexities and variations in transnationalism between and within groups and by generation and through generational choices relating to community engagement and geographical proximity. It has shown how refugee backgrounds do make a difference to modes of transnational engagement and the reasons why the second generation from refugee backgrounds may, or may not, be transnational. To take a transnational perspective, as Dahinden (2017) argues, it is necessary to locate any analysis within both a national and de-national framework. The comparative approach used in this paper enables a more nuanced understanding of the multiple and variable impacts of the specificities of biography and of structure and the complex ways in which they interact bringing new insights into the analysis.
